# Emerging ESG reporting of higher education institutions in China

**DOI:** 10.1016/j.heliyon.2023.e22527

**Published:** 2023-11-19

**Authors:** Fei Mo, Derek D. Wang

**Affiliations:** a School of International Organizations, Beijing Foreign Studies University, 19 Xisanhuan North Road, Beijing, 100089, China; b School of Business Administration, Capital University of Economics and Business, 121 Zhangjialukou, Beijing, 100070, China; c The China ESG Institute, Capital University of Economics and Business, 121 Zhangjialukou, Beijing, 100070, China

**Keywords:** ESG, Sustainability reporting, Higher education institutions, China

## Abstract

This paper investigates the ESG reporting practices of Chinese higher education institutions (HEIs). Based on Global Reporting Initiative's common reporting standards and the education sector-specific indicators proposed by earlier studies, we construct a disclosure framework of 112 indicators classified into environmental, social, governance, and educational dimensions. We manually collect all ESG-related information disclosed by 147 elite Chinese HEIs from various channels, including 8 standalone sustainability reports, around 600 annual reports, 139 charters, official websites, and official social media accounts, for the period 2020–2023. The results demonstrate how Chinese HEIs disclose different types of ESG information via different channels, with most of the information disclosed through channels other than standalone sustainability reports. Most Chinese HEIs do not adopt any specific disclosure standards but loosely follow the Sustainable Development Goals (SDGs) in reporting. We further assess the quality of the disclosed information for each indicator and observe that, quality-wise, *governance > educational* > *social* > *environmental*, highlighting the necessity for Chinese HEIs to enhance environmental reporting. Next, we investigate the determinants of reporting quality and find that an HEI's number of faculty members is a more significant factor associated with its ESG reporting quality than other factors (i.e., number of students, funding, expenditure). Chinese HEIs are advised to report in a more balanced manner across the four dimensions in a standalone report using an education-oriented indicator-based framework instead of the current SDGs-based framework, to improve the completeness, materiality, and comparability of information.

## 1 Introduction

Higher education institutions (HEIs) play a unique multi-faceted role in human society's efforts to achieve sustainable development [1]. The worldwide higher education sector, as a behemoth with over 88,000 HEIs and 235 million students, can lead to negative environmental and social consequences if proper measures are not taken [2]. HEIs face numerous challenges in this regard, including issues such as energy consumption, air pollution, waste management, student safety, community relations, diversity, equity, and inclusion [3]. Nevertheless, HEIs can leverage their roles as educational and innovative organizations to foster sustainable practices through research that creates sustainability knowledge, teaching that cultivates current and future sustainability implementers, and community outreach that nurtures cross-sectoral sustainability collaborations [4]. Consequently, enhancing the sustainability of HEIs has long been recognized as a critical task, as evidenced by the formation of a series of high-profile international declarations, charters, and networks [5].

The reporting of environmental, social, and governance (ESG) information is considered crucial for achieving sustainability objectives and engaging various stakeholders [6]. Although currently focused primarily on corporate entities, there is growing recognition that HEIs should disclose ESG information as well [7]. Multiple theoretical frameworks offer explanations for why HEIs choose to release ESG information, including legitimacy theory, stakeholder theory, signaling theory, and institutional theory [8]. Legitimacy theory points out that organizations must obtain legitimacy to continue operating within society. Given society's increasing awareness of sustainability issues, ESG reporting can aid organizations in attaining legitimacy. On another note, stakeholder theory advocates for organizations to act in the interests of a wide range of constituents beyond just shareholders. As pointed out in Ref. [8], “other than traditional financial reporting, which largely caters to shareholder's information needs, sustainability reporting (supposedly) offers valuable information to a broader audience.” Signaling theory proposes that an entity can leverage reporting to address information asymmetry related to sustainability concerns and bolster its reputation through sharing verifiable details with key parties. Institutional theory highlights how an entity will often behave based upon prevailing industry norms and anticipated expectations. By embracing sustainability reporting, firms can demonstrate conformity to these broader expectations.

A growing stream of research has examined HEIs' sustainability reporting since early 2000s. Most of the studies in this field strive to illuminate the characteristics of HEIs' reporting practices, such as their extent and informativeness, by benchmarking the reported ESG information to established standards [[9], [10], [11]]. The Global Reporting Initiative (GRI) standards, and their variants, are widely used as the framework to report and assess ESG information released by HEIs [7]. The newest version of GRI framework includes three series of standards: GRI Universal Standards applicable to all organizations, GRI Sector Standards applicable to specific sectors, and GRI Topic Standards for material topics if they are applicable to the reporting organization [12]. Right now, GRI has not released the sector standards for the education sector. The GRI Universal Standards require disclosure of information on the reporting organization's activities, governance, and policies, and set forth the guidelines on determining the material topics. The GRI Topic Standards classify sustainability indicators into economic, environmental, and social dimensions. Since HEIs shoulder the responsibility of educating the next generation of professionals and decision makers who shape society, it is generally accepted that the GRI Standards should be augmented with the educational dimension [13].

Despite intensifying public concern about sustainability, the adoption of ESG reporting practices among HEIs remains low. For example, the Sustainability Tracking, Assessment & Rating System (STARS), the largest online self-reporting portal for HEIs, lists 1,158 registrants as of June 2023, accounting for only 1.32 % of the 88,000 existing HEIs worldwide. Many submissions lack accuracy or coherence. Hence, from a global perspective, HEIs’ ESG reporting is at an early stage. A clearer understanding of hindrances and propellers behind ESG reporting would likely promote further development on several fronts simultaneously.

This study investigates the online ESG reporting practices of 147 leading and highly esteemed universities in China between 2020 and 2023. Due to China hosting the world's greatest population of college students, understanding how these institutions communicate their ESG activities holds considerable significance. Our research examines various sources like standalone sustainability reports, annual reports, university charters, webpages, and social network posts, to gather data. We assess the disclosed information based on textual and content analysis under a framework of 112 ESG indicators adapted from the GRI standards and earlier literature. We evaluate the quality of HEIs' disclosure and explore the determinant factors.

This study makes four contributions. First, it seeks to enhance knowledge of the ESG reporting landscape of HEIs in China. Overall, systematic ESG reporting is still in its infancy in China, with only 4% of sampled HEIs publishing standalone sustainability reports, all of which are in Beijing or Shanghai. Chinese HEIs tend to disclose ESG information in a sporadic, fragmented, and impromptu way, as pieces of related information is reported through different channels intermittently. Second, we find that Chinese HEIs almost invariably organize ESG information in their standalone reports around the 17 Sustainable Development Goals (SDGs) of the United Nations, without specifying any target or indicator. However, the SDGs are not designed for the education sector, and may impair the materiality, accuracy, and comparability of HEIs’ disclosed information. Third, in terms of reporting quality, *governance > educational* > *social* > *environmental*. Chinese HEIs are therefore advised to improve their reporting of environmental information. Finally, it investigates the determinants of reporting practices and finds that the number of faculty members is a more significant factor associated with reporting quality than number of students, funding, and expenditure.

The remainder of the paper proceeds as follows: Section 2 surveys the literature, Section 3 presents the research design, Section 4 presents the results, Section 5 discusses the findings, and Section 6 concludes.

## 2 Literature review

This study is at the intersection of two fields of study: ESG information disclosure and HEIs’ sustainability. The literature on ESG information disclosure comprises a substantial body of papers that have explored a wide spectrum of topics, including the determinant factors of disclosure [14], the consequences of disclosure [15], the regulations and standards [16], and the quality and informativeness of disclosure [17]. Academic papers often draw on legitimacy theory, stakeholder theory, signaling theory, and institutional theory to explain the motives behind sustainability reporting [8]. We note that most papers in this field deal with disclosure by the corporates.

HEIs' sustainability problem has received increasing attention since early 2000s. Numerous papers have studied a broad set of sustainability issues associated with HEIs, including strategic sustainability planning [18], sustainability activities such as renewable energy use [19], sustainability education [20], and sustainability assessment and evaluation [[21], [22], [23]]. Prior research notes that HEIs differ from firms as they shoulder the critical responsibility of cultivating future professionals/leaders and creating new knowledge [2,3]. Therefore, it is generally agreed that HEIs’ sustainability measures should not be limited to their own sustainable operations, such as pollution control and waste reduction, but should also include sustainability education and innovation based on their core functionality in society [13,24].

A vast body of papers study HEIs' sustainability reporting. The literature in this area can be classified into two categories: (1) analysis and assessment of reporting practices, including the status, trends, causes, consequences, and relationships with other factors, and (2) development of reporting tools and standards. In the first category, a significant number of papers have empirically analyzed HEIs’ sustainability reporting practices in different countries, including German and Austrian HEIs [25], Canadian HEIs [26], Ghanaian HEIs [27], Italian HEIs [11,28], United Kingdom HEIs [29], Australian HEIs [10], and the United States HEIs [30]. The focus of analysis includes the adequacy, extent, and determinants of disclosure. However, most papers in this area exclusively analyze the voluntarily disclosed information in standalone sustainability reports and ignore ESG information disclosed via other channels (e.g., annual reports, charters, social media).

A significant amount of effort is devoted to developing reporting standards and tools for HEIs. The most widely used reporting standard for HEIs is the GRI Standards [12]. Since the GRI Standards are designed for all sectors, some variants have been developed to match the specific needs and accommodate the characteristics of the higher education sector. Ref. [31] develops a graphical tool Graphical Assessment of Sustainability in Universities (GASU) that assesses information disclosed in accordance with the GRI Standards. Another work [32] develops an ESEG assessment tool for HEIs, where the second “E” stands for education. Another popular reporting and assessment framework is the STARS developed by the Association for the Advancement of Sustainability in Higher Education (AASHE) [33]. STARS is a transparent, self‐reporting online framework that categorizes HEIs’ sustainability information into four dimensions: academics, engagement, operations, and planning & administration.

Finally, we note that a couple of studies have examined sustainability reporting by Chinese HEIs. Ref. [34] analyzes the characteristics of sustainability reports collected from a government-led initiative in China between 2012 and 2016, covering 118 reports from 44 HEIs in two provincial-level administrative regions. However, the reports are submitted to the local governments through a mandatory one-time program but not released to the public. Hence the sample does not reflect the landscape of voluntary information disclosure in the whole country. Another study [35] examines online disclosure by 70 Chinese HEIs from seven regions defined by the authors, with the focus on regional comparisons. Overall, the extant studies on Chinese HEIs are limited in several ways. First, the sample of HEIs is biased. Second, the information collection is restricted to a single channel and fails to account for information disclosed across diverse channels. Third, the existing works neglect the determinant factors behind reporting. Consequently, it is still unclear how Chinese HEIs report ESG information and how their reporting practices can be improved. This study addresses the research gap by offering a comprehensive evaluation of the latest online contents of 147 leading Chinese HEIs and explores the determinants of their reporting practices.

## 3 Research design

### 3.1 Method

**Fig. 1:**
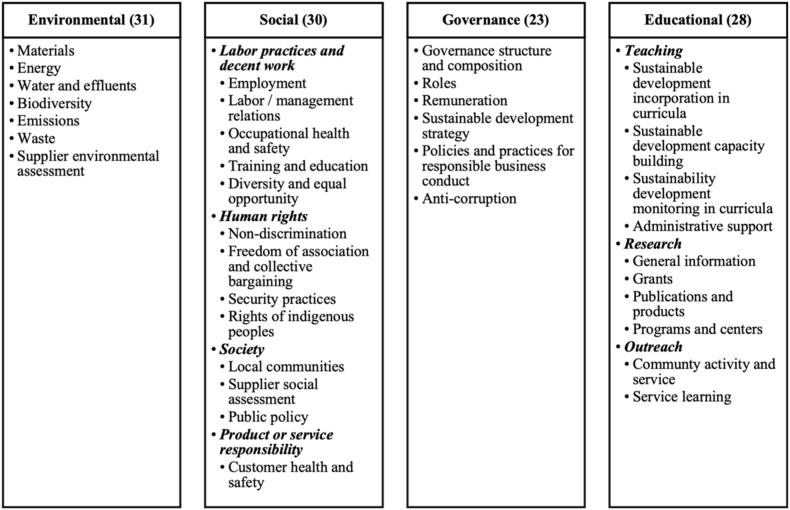
Categories and aspects of indicators.

This study applies a content analysis method based on ESG indicators. We combine the GRI common reporting standards with the educational component of GASU proposed in Ref. [31], and classify the indicators into four dimensions: environmental, social, governance and educational. The categories and aspects of the indicators used are presented in Fig. 1. The full set of indicators are in Tables A1 to A4 in the Appendix. We make necessary decisions as to what indicators to include. For GRI Universal Standard 2 “General Disclosures 2021”, we exclude Disclosures 2–1, 2-2, 2–3, 2–4, 2–5, 2–6, 2–7, 2–8, 2–29, or 2–30, because they refer to generic information that almost all HEIs adopt the same approach as to their online reporting. For GRI Topic Standards, we exclude GRI 201, 202, 203, 204, 206, 207, 408, 409, 417, and 418, because none of them is a material topic for HEIs. This leaves us with 112 indicators, including 31 environmental, 30 social, 23 governance, and 28 educational. The detailed requirements of the indicators belonging to the environmental, social, and governance dimensions can be found in the latest consolidated set of GRI standards [12]. For each indicator, we parse and evaluate the HEIs' disclosed contents using a scale of 0–4, where 0 means a total lack of information, 1 means the information disclosed equates to around 25 % of full disclosure for this indicator, 2 means 50 %, 3 means 75 %, and 4 an excellent 100 %. We sum up the scores for each indicator to obtain the dimensional reporting scores and the overall reporting scores for a single HEI and eventually the overall reporting quality of all HEIs. A similar content scoring method has been employed in early studies on HEIs’ disclosure [32].

We investigate the determinants of reporting quality through ordinary least squares (OLS) regression as follows,

(1) $${ReportingScore}_{i}=\beta_{0}+\beta_{1}{Student}_{i}+\beta_{2}{Faculty}_{i}+\beta_{3}{Funding}_{i}+\beta_{4}{Expenditure}_{i}+Province\;Dummies+\epsilon_{i}\text{.}$$

In (1), i labels an HEI and we include variables that may affect the HEI’s reporting practices. Reporting practices may depend on the HEI's size since larger HEIs may face greater pressure on sustainability and have more resources to carry out reporting. Variables Student and Faculty represent, respectively, the number of students and the number of faculty members. Reporting may also depend on the HEI's financial status. We include Funding from all sources measured in million RMB, and Expenditure measured in million RMB. Finally, we include ProvinceDummies to control province-fixed effect since reporting score may be affected by local policies and socio-economic situations.

### 3.2 Data

There are a total of 3,012 HEIs in 2021 in China [36]. This study focuses on 147 elite HEIs admitted into the “Double First-Class” program in China. “Double First-Class” is a higher education initiative launched by the Chinese government in 2015, with the aim to foster world-class universities and disciplines and enhance the country's competitiveness in science and technology [37]. As the overarching program for elite higher education, “Double First-Class” supersedes two predecessors, “Project 985” and “Project 211” initiatives. Our sample consists of the 147 HEIs on the latest list of the Double First-Class initiative [38] (Table A5 in the Appendix).

All sampled HEIs are public institutes affiliated to and funded by either the Ministry of Education of China (MOE), a ministry or committee of the central government, a provincial-level administrative region, a provincial-level bureau of higher education in China, or certain special association or public institution [39]. Regardless of their affiliation, Chinese HEIs are governed by national and local laws and regulations, if applicable. Article 44 of the Law on Higher Education imposes an obligation on Chinese HEIs to disclose information on the evaluation of its operations and quality of education for purposes of supervision by the society. The scope of the information that HEIs must disclose is specified by a 2010 MOE regulation, including sustainability-related items such as HEI internal management and leadership information, charter and procedures for hearing and handling student complaints [40]. The regulation in Articles 8, 12 and 14 also asks HEIs to publish a catalogue of such information and any additional information it voluntarily discloses and to publish via a media such as the HEI's official website, and update information in time [40].

This study focuses on online information disclosure. We note that offline ESG disclosure may exist in the form of internal report, but offline information is mostly inaccessible by the public. Preliminary investigation informs us that Chinese HEIs can disclose information online through diverse channels in various formats. We conduct extensive and exhaustive search on the HEIs’ websites and social media accounts with the following keywords: report; annual report; sustainability report; ESG report; SDG report; sustainable development; ESG; SDG; green; climate; governance; social responsibility; office of sustainability; and charter in both Chinese and English. If necessary, we carry out individual searches on the respective websites and social media accounts for additional information. We restrict our attention to 2020–2023 since pre-2020 disclosure by the sampled HEIs is rare.

We identify five channels through which Chinese HEIs disclose ESG information: standalone sustainability reports, annual reports, charters, webpages, and social media posts. If an HEI releases multiple sustainability reports in 2020–2023, all of them are included. The reports focusing on a single sustainability dimension are included. Reports issued by a particular school of an HEI are also included. Besides, we download the charter of HEIs if there is one. An HEI can report through multiple channels. The information we retrieve for an HEI is referred to, collectively, as the online contents of the HEI.

## 4 Results

### 4.1 General reporting status

**Fig. 2:**
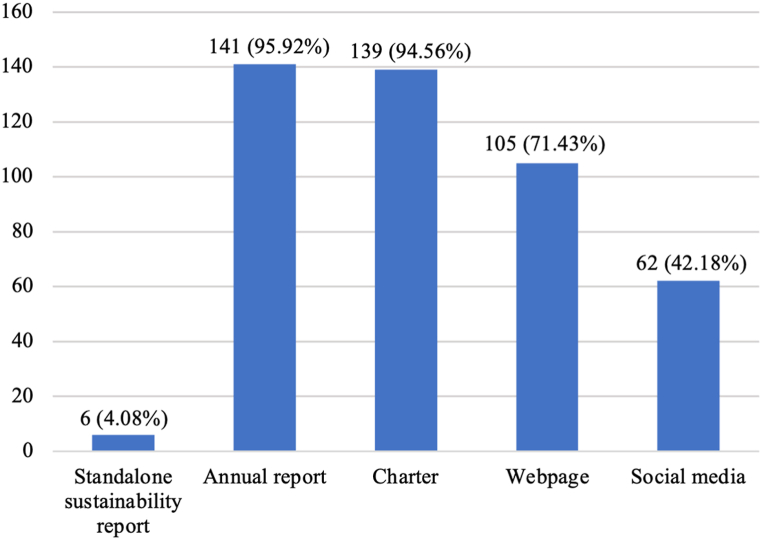
Disclosure channels used by HEIs (Note: the vertical axis is the count of HEIs).

**Fig. 3:**
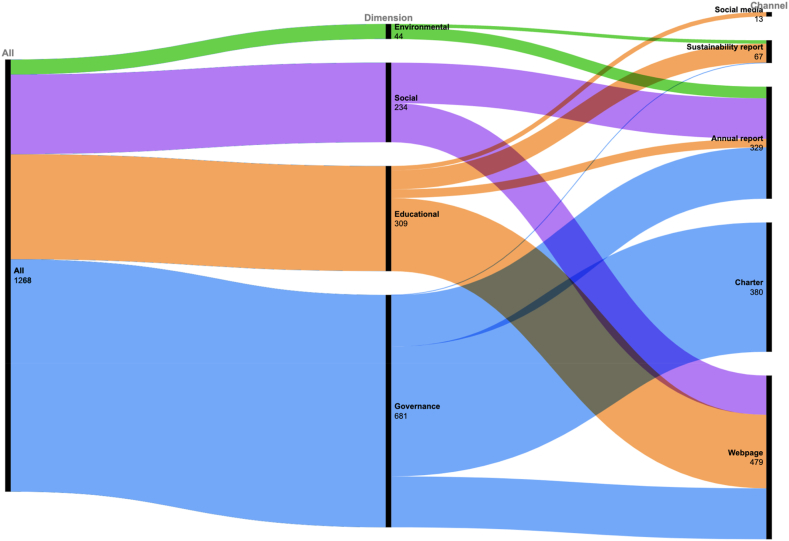
Distribution of reported indicators in different dimensions and channels (Note: the number indicates the sums of indicators that all HEIs report in a specific dimension or channel.).

Fig. 2 depicts the disclosure channels used by the HEIs. Annual report is the most widely used channel and standalone sustainability report is uncommon. Few HEIs release standalone sustainability reports and the ones published are inconsistent across HEIs in several key aspects. Fig. 3 illustrates the distribution of reported indicators across different dimensions and channels. The HEIs report on 1,268 indicators (i.e., 1,268 HEI-indicator observations) with governance indicators constitute more than half of them. Webpage is the most used channels with 479 HEI-indicators from social, educational and governance dimensions. The charter channel contains only governance information. The social media channel only contains educational information. Annual report contains information from all four dimensions. Environmental information is disclosed via sustainability report and annual report. Social media only contains educational information. Standalone sustainability report lacks information in the social and governance dimensions.

In total, 4 % (6 of 147) of the sample HEIs publish standalone sustainability reports (Table 1). All 6 HEIs are in Beijing or Shanghai. We see great variance in terms of the type and publisher of the reports: the management schools of 2 HEIs publish school-level reports focusing only on environmental and educational dimensions [[41], [42], [43]]; 1 HEI issues a report solely on educational dimension about its sustainability research performance [44]; the remainder of the reports are published by an office of planning, an office of international affairs or an institute for sustainability and contain HEI-level sustainability information on all four dimensions [[45], [46], [47], [48]]. Only 1 report contains a statement by the school dean [43]. Fudan University also makes accessible supporting documents for its report [49]. In most cases, the reports are available in both Chinese and English (67 % or 4 of 6). Tongji University issues its report only in English [44].

**Table 1:** Details of the standalone reports by Chinese HEIs.

No.	HEI Name	Provincial Region	Report Name	Languages	Date of Publication	Reporting Year	Pages	Reporting Framework	Coordinator or Publisher	Leadership Statement	Audit
1	Peking University	Beijing	Carbon Footprint Report (2021)	Chinese, English	26-Feb-22	2020–2021	12	ISO14064-1	Guanghua School of Management	No	n.a.
			Carbon Footprint and Carbon Neutralization Action Report 2022		3-Apr-23	2021–2022	13			No	n.a.
2	Tsinghua University	Beijing	Report on Sustainable Development Goals	Chinese, English	28-Apr-21	2020–2021	88	n.a.	Office of International Affairs Office of Hong Kong, Macao and Taiwan Affairs & Institute for Sustainable Development Goals	No	n.a.
3	Fudan University	Shanghai	SDGs Action Report	Chinese	23-Nov-21	2016–2020	71	n.a.	Office of Planning	No	n.a.
4	Tongji University	Shanghai	Assessing universities' contributions to Sustainable Development Goals: An example from Tongji University on its research performance	English	20-May-22	2002–2020	65	n.a.	Tongji University & Elsevier	No	n.a.
5	Shanghai Jiao Tong University	Shanghai	GHG Emissions Report and Climate Action (2021–2022)	Chinese, English	6-May-23	2021–2022	33	ISO14064-1	Antai College of Economics and Management & Chamwion Tech	Yes	Yes
6	Shanghai University	Shanghai	SDGs Action Report	Chinese	25-Oct-21	2020–2021	60	n.a.	Office of Strategic Development and Planning	No	n.a.
					11-Nov-22	2021–2022	55			No	n.a.

The results point to lack of disclosure framework, reporting continuity and external assurance for ESG reporting by Chinese HEIs. Peking University and Shanghai Jiao Tong University use the ISO14064-1 standard to report their carbon emissions, while the others do not base their reports on any standard, but all choose to organize their information around the 17 SDGs of the United Nations, without specifying any target or indicator. The sustainability reports are between 12 and 88 pages in terms of length (50 pages on average). All reports are released after 2021. Peking University and Shanghai University publish reports annually. Others have only published once. For 6 of the 8 reports, the reporting period is the year right before the year in which the report is published. The dates of publication are not specified in the reports but can be verified by referring to the webpages containing the links to the reports or to the online press releases for the reports. Two HEIs collaborate with external sources when preparing their reports: Tongji University collaborates with Elsevier, the Dutch academic publishing company providing research analytics among others; Shanghai Jiao Tong University collaborates with Chamwion Tech, a Chinese ESG service provider that specializes on ESG reporting, carbon neutrality and climate risk analysis. Shanghai Jiao Tong University seeks external assurance from Shanghai Center for Energy Saving and Emission Reduction for the compliance of its report with ISO 14064–1 and related standards [43].

Chinese HEIs publish a variety of annual reports that contain ESG information. As to other online contents of HEIs, most HEIs (96 % or 141 of 147) make available their public disclosure portals with information disclosure catalogs, others either do not have any public disclosure portal or do not make such information publicly accessible. Only 2 HEIs (Shanghai Jiao Tong University and Xiamen University) publish HEI-level comprehensive annual reports. Other HEIs publish a number of topic-specific annual reports. Among the different types of annual reports published within the reporting period, information disclosure annual report is the most common one (96 % or 141 of 147), followed by undergraduate teaching quality report (93 % or 136 of 147), employment quality report (75 % 110 or of 147), art education report (45 % or 66 of 147), faculty and union conference report (35 % or 51 of 147), and academic committee report (29 % or 43 of 147). Certain HEIs also publish graduate education quality annual report (10 % or 14 of 147) and scientific research results report (2 HEIs). Some HEIs also make available law-based governance report (9 % or 13 of 147), energy and resource saving management report (8 % or 12 of 147) and food safety report (1 HEI), and it should be noted that these three types of reports are only disclosed by HEIs in Shanghai.

Aside from annual reports, most HEIs (95 % or 139 of 147) release their charters to the public. A considerable number of HEIs publish extensive sustainability-related contents on their websites (71 % or 105 of 147) or via social media platforms such as WeChat or Weibo (42 % or 62 of 147). It should be noted that some of the reports and charters cannot be downloaded and can only be viewed online.

**Fig. 4:**
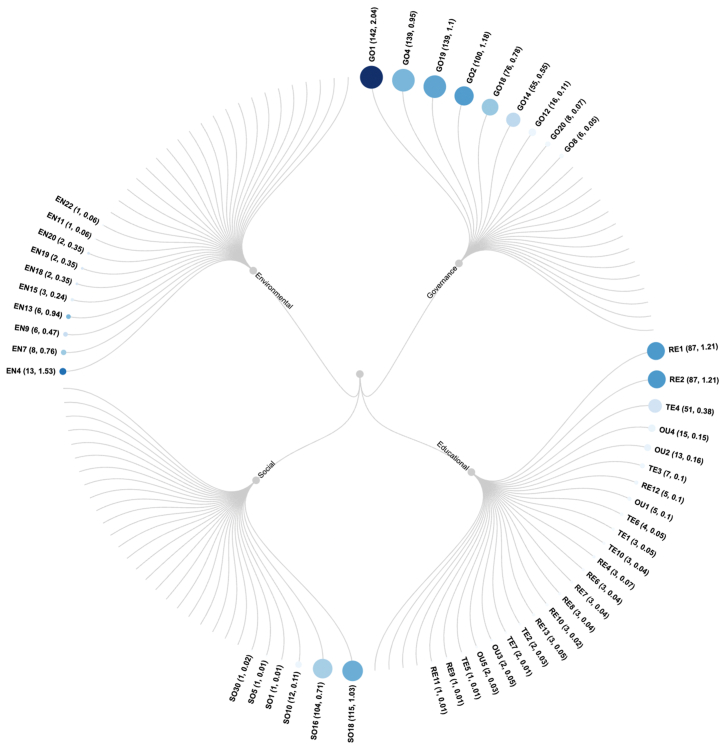
Overview of indicator-wise reporting results.

Fig. 4 presents an overview of the indicator-wise reporting results. We observe that the social dimension features the fewest indicators with HEIs’ reporting, and the educational dimension features the most indicators with reporting. If we sum up the reporting HEIs across all indicators under a specific dimension, we see that the environmental dimension has the fewest HEIs reporting on its indicators and the governance dimensional has the most HEIs reporting. The indicator GO1 exhibits the highest reporting score. Below we analyze the results across different dimensions in details.

(Note: Each leaf node represents an indicator. Size of the leaf node represents the number of HEIs that report on this indicator (the first number in parentheses). Color of the leaf node represents the reporting score (the second number in parentheses; the darker, the larger). Labels for indicators without reporting are not shown. Please refer to the **Appendix** for definitions of the indicators.)

### 4.2 Environmental dimension

Environmental reporting by Chinese HEIs is generally poor. Only a small fraction of HEIs (12 % or 17 of 147) report on the environmental dimension (Table A6 in the Appendix) and all of them are in either Beijing (3 HEIs) or Shanghai (9 HEIs). The average reporting level is only 1 %, the lowest of all four dimensions. 10 of 31 indicators are used. They used on average 3 indicators. No indicator reaches an above-average level ( ≥ 2.0 points). The total scores the HEIs received are between 1 and 16 points. Shanghai Jiao Tong University's online disclosure is the most comprehensive in the environmental dimension (13 %).

The sampled HEIs do not report on materials, waste, and supplier environmental assessment. Only 2 HEIs provide information on biodiversity, specifically on the number and type of hardy plants cultivated on campus and the improved campus aquatic biodiversity in the sustainability report and the faculty and union conference report, respectively [48,50]. The sampled HEIs perform slightly better in terms of energy, water, and emissions. Out of the 15 HEIs located in Shanghai, 14 report on their energy consumption (EN4), 6 on reductions in energy consumption (EN7), and 8 on interactions with water (EN9), water withdrawal (EN11) and water consumption (EN13) in their energy and resource saving management reports. Tsinghua University in Beijing is the only HEI reporting on energy and water outside Shanghai, disclosing the amount of reductions in energy consumption in its sustainability report [45]. Peking University and Shanghai Jiao Tong University are the only HEIs reporting on emissions, covering direct and indirect GHG emissions at the school level [[41], [42], [43]]. All 3 reports (2 by Peking University) are accompanied by a carbon reduction action plan or a net zero plan, but those plans do not set quantified emission reduction or energy consumption reduction targets for the HEIs [[41], [42], [43]].

It is worth noting that 26 of 147 HEIs (18 %) expressly state environmental sustainability goals in their charters, such as building a green or ecologically friendly campus or protecting the historic buildings and old trees on campus. Those HEIs are in 14 different provincial-level administrative regions. We discuss their expressions further in the governance dimension section below. Only 4 universities, all located in Beijing or Shanghai, state environmental sustainability goals as well as report on the environmental dimension. Other HEIs with environmental sustainability goals simply forgo reporting on environmental issues.

### 4.3 Social dimension

A quite sizable number of HEIs (93 % or 136 of 147) report on the social dimension (Table A7 in the Appendix). But they use a very limited number of indicators (6 of 30) and report at a low level (2 %). The received in total between 1 and 5 points. Peking University and Beijing Jiaotong University report most comprehensively in this regard (5 points or 4 %), while 9 HEIs receive 4 points, 19 HEIs 3 points, 67 HEIs 2 points and 39 HEIs only 1 point.

The 6 indicators belong to labor-practices (5 indicators) and product or service responsibility (1 indicator). Indicators within the sub-categories of human rights and society are not reported at all. No indicator surpasses the average level ( ≥ 2.0 points). The indicator SO18 (diversity of governance bodies and employees) achieves the highest score (1.03 points). 78 % (115 of 147) of the sample HEIs provide information on this indicator. 13 HEIs (9 %) make available both the gender and ethnic group information of their governance body members on a single webpage, two HEIs only the gender information, and 1 HEI only the ethnic group information (but with photos of the individuals which may allude to their gender). Another 15 HEIs (10 %) make available the photos of their governance body members, which may allude to their gender. A large number of HEIs (71 % or 104 of 147) report on the percentage and/or number of employees by age group in their undergraduate teaching quality reports, although 19 of them disclosed only the number or percentage of employees under 45 or 40.1 HEI even provides employee age break-downs at the school level [51]. Only 3 HEIs report on the number of employees by gender: Beijing University of Technology and Shanghai International Studies University report on their websites and the data for Beijing University of Technology dates back to 2016 [52,53]; Shaanxi Normal University report in its undergraduate teaching quality report and is the only HEI giving employee break-downs both by gender and by age groups [54].

69 % (101 of 147) of the sampled HEIs provide information on upgrading employee skills (SO16) in their undergraduate teaching quality reports and/or faculty and union conference reports. But the coverage is limited to the training programs provided to junior faculty members, obtaining only 1 point for those HEIs. A small number of HEIs also report on new employee hires and employee turnover (SO1, 1 HEI receiving 1 point), occupational health and safety (SO5, 1 HEI receiving 1 point), promotion of worker health (SO10, 12 HEIs receiving on average 1.3 points) and non-compliance regarding health and safety impacts of services (SO30, 1 HEI receiving 3 points). For the last indicator, Fudan University publishes on its portal food safety reports containing the results of third-party testing and laboratory testing of the environment and the products in student canteens, and provides reasons and corrective measures for any failure to pass the test.

### 4.4 Governance dimension

Almost all HEIs (98 % or 144 of 147) report on the governance dimension (Table A8 in the Appendix) at a reporting level of 7 %, highest among the four dimensions. The HEIs use 9 of 23 indicators, and they used on average 5 indicators (spanning from 1 to 7). Tongji University received the highest score with 16 of 92 points (17 %).

Out of the 9 governance indicators, the indicator GO1 (governance structure and composition) reaches an above-average level ( ≥ 2.0 points) with 2.04 points. 142 HEIs (97 %) described their governance structure on their websites, among which 16 received additional points for describing the gender and ethnic group information of the highest governance body members. The HEIs report on other governance indicators with a combination of charters and annual reports. In their charters, HEIs provide information on nomination and selection of the members of the party committee, the highest governance body (GO2, 100 HEIs or 68 %), the role of the highest governance body (GO4, 139 HEIs or 95 %), the communication of critical concerns (GO8, 6 HEIs or 4 %), the remuneration policies (GO12, 16 HEIs or 11 %), and the statement on sustainable development strategy (GO14, 55 HEIs or 37 %). Among the 55 HEIs with statements on their sustainability development strategy, 26 expressly refer to “sustainable development”. Dimension-wise, statements from 12 HEIs only cover environmental sustainability goals, i.e., ecologically-friendly or green campus, 2 HEIs only refer to social goals, i.e., trainings, skills development, safeguarding, motivation and supervision in favor of the sustainable development of faculty and staff, 12 HEIs only contain governance goals, focusing on planning and efficient use of funds and resources, 10 HEIs only have educational goals, e.g., providing service, talent and support to the construction of ecological civilization or sustainable development, constructing a coordinated and sustainable system of disciplines, and 19 HEIs mention more than one dimensions.

76 HEIs (52 %) describe in their charters the mechanisms for seeking advice and raising concerns (GO18). Additionally, a specific section of the law-based governance reports of 12 HEIs in Shanghai report comprehensively on this indicator. 139 HEIs (95 %) in their information disclosure reports report on compliance with laws and regulations in terms of information disclosure (GO19), and only 1 HEI admits having been involved in a case, but without giving any detail [55]. Additionally, a specific section of the law-based governance reports of 12 HEIs in Shanghai give detailed information on the numbers and details of the different types of cases they are involved in. 8 HEIs (5 %) describe their membership associations (GO20) on their websites and in their sustainability reports (Tsinghua University and Fudan University), featuring collaboration with foreign HEIs and international organizations and established mainly after 2018 (Table 2) [45,46,[56], [57], [58]].

**Table 2:** Membership associations of certain Chinese HEIs.

No.	HEI Name	Provincial Region	Membership	Description	Since	Source(s) of Information
1	Peking University	Beijing	University Alliance for Sustainability (UAS)	An alliance of 5 HEIs from five different countries started by Freie Universität Berlin to promote collaboration in sustainability research, teaching and campus management	2015	Website
2	Tsinghua University	Beijing	Global Alliance of Universities on Climate (GAUC)	An alliance of 13 HEIs from nine countries focusing on climate-related joint research, student activities, talent training, green campus and public engagement	2019	Sustainability report; Website
3	Communication University of China	Beijing	International Academic Network for a Community with Shared Future (IAN-CSF)	A network of Institute for a Community with Shared Future of the Communication University of China and 16 international research centers in 15 countries	2019	Website
4	Fudan University	Shanghai	FD-QM Online Course Quality Standard Alliance	An alliance of more than 60 Chinese HEIs for the formation of online course quality standards comparable to international standards	2018	Sustainability report
			Yangtze River Delta University Alliance for Sustainability	An alliance of 8 Chinese HEIs to promote cooperation in sustainability teaching, research, social services, international communications and advisory	2021	Website
5	Tongji University	Shanghai	Yangtze River Delta University Alliance for Sustainability	Same as above	2021	Website
6	Southeast University	Shanghai	Yangtze River Delta University Alliance for Sustainability	Same as above	2021	Website
7	Shandong University	Shandong	UNIDO “Clean Energy Innovation” University Alliance	An alliance of UNIDO and a group of Chinese HEIs to strengthen international exchanges and cooperation to achieve UN's clean energy sustainable development goals	2023	Website
8	Sichuan University	Sichuan	UNIDO “Clean Energy Innovation” University Alliance	Same as above	2023	Website

### 4.5 Educational dimension

A majority of HEIs (68 % or 100 of 147) report on the educational dimension (Table A9 in the Appendix) at a reporting level of 4 %. The HEIs vary in terms of reporting quality. Their scores range between 1 point and 60 points (54 %) (Fudan University). They used on average 2 indicators and 24 of the 29 indicators were used. No indicator surpasses the average level ( ≥ 2.0 points). The indicators RE1 (presence of research in sustainable development) and RE2 (list research issues addressed) achieves the highest score with 1.21.

52 HEIs (35 %) report on the teaching category with a reporting level of 2 %, lowest of all three categories of the educational dimension. The low level of reporting in this category is probably related to the fact that most reporting HEIs are disclosing sustainability teaching information on their websites instead of in separate reports, therefore making such information scattered and incomplete. The indicator TE4 is reported most comprehensively, with 50 HEIs disclosing sustainability-related course titles and themes contained, but such disclosure is by no means complete. We see regional concentration of those reporting HEIs: 29 HEIs or 58 % are from Beijing (13), Shanghai (6), Nanjing, Jiangsu (5) and Chengdu, Sichuan (5). This might suggest promotion of sustainability curriculum at the local level. The indicators TE8 (management structure) and TE9 (administrative support) are not used at all, probably suggesting that sustainability teaching do not receive enough attention from the Chinese HEIs’ management and top-down administrative effort in monitoring and supporting sustainability curriculum is lacking.

87 HEIs (59 %) report on the research category with a reporting level of 5 %, highest of the three categories of the educational dimension. The relatively high reporting level of the research category might be related to the strong research focus of the “Double First-Class” program, according to which the sampled HEIs were selected [37]. HEIs with standalone reports performed the best in this regard, providing the knowledge fields, issues, faculty names and summaries of as well as institutional support to their sustainability-related research, and Tongji University's report focused exclusively on research [[41], [42], [43], [44], [45], [46], [47], [48]]. Other HEIs mainly used their websites and sometimes also their social media accounts to disclose information on sustainability-related research. The indicators RE3 (percentage of relevant graduate students) and RE5 (percentage of relevant faculty) were not covered by any HEI, probably because they would need input from all schools and institutes of the HEIs, no matter how remotely related they are to sustainability. Collecting research highlights from sustainability-oriented schools and institutes appears to be a much easier task.

Only 23 HEIs (16 %) report on the community outreach category, but they collectively use all 5 outreach indicators and had the second highest reporting level (2.4 %). The indicators OU2 and OU4 of the outreach category see a high number of references, 13 and 15, respectively, in their standalone reports and undergraduate teaching quality reports and on their websites. If we take a regional view of these two groups of reporting HEIs, we can see that about half of both groups of HEIs are located in Beijing and Shanghai, and in terms of the other half, HEIs from the coastal provinces (Jiangsu, Fujian and Guangdong) are more prone to disclose information on partnerships for sustainability while HEIs from the northeast provinces (Liaoning, Jilin and Heilongjiang) and southwest and northwest provinces (Sichuan, Guizhou and Shaanxi) are more informative on service learning programs.

HEIs tend to disclose more information on qualitative indicators than on quantitative indicators in the educational dimension. We may divide the 28 educational indicators into 17 qualitative (TE4, TE5, TE6, TE7, TE8, TE9, RE1, RE2, RE4, RE6, RE7, RE8, RE10, RE12, OU1, OU2 and OU4) indicators and 11 quantitative indicators. We note that 99 HEIs report on qualitative indicators while only 7 HEIs report on quantitative indicators. This might suggest that, despite many HEIs disclosing general information on or best practices of educational indicators, HEIs lack the will or tool to collect and disclose exact data which allows a year-on-year comparison and shows the overall progress towards sustainable education goals in their institutions.

### 4.6 Evaluation

**Fig. 5:**
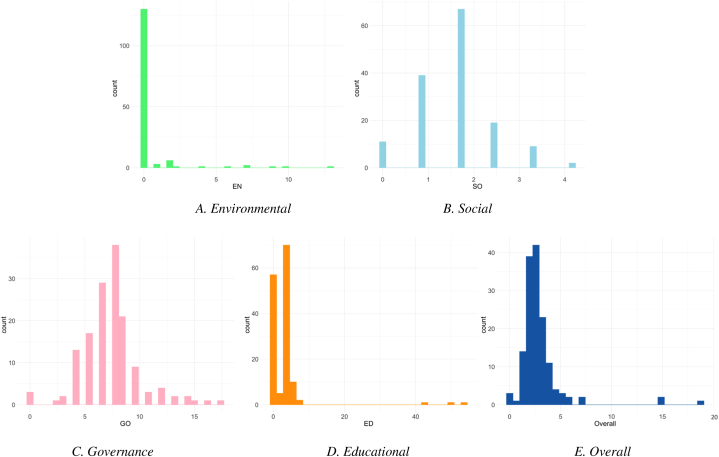
Histograms of reporting levels **(**Note: vertical axis shows count of HEIs, horizonal axis shows reporting levels in percentage**)**.

Fig. 5 summarizes the distribution of reporting levels in all four dimensions. In terms of the environmental and educational dimensions, reporting levels are concentrated at the lower end with a significant portion of HEIs at 0 or close to 0 and a few outliers at the upper end. For the social and governance dimensions, the distribution of reporting levels is bell-shaped. The results show an uneven disclosure landscape in terms of the four dimensions: Almost all sampled HEIs include information on social and governance dimensions, about two thirds cover the educational dimension, but only 17 disclose information on environmental dimension. 13 HEIs or 9 % take all four dimensions into account and all of them are in Shanghai or Beijing. Overall, we can confirm from *t*-test that *governance* ≻ *educational* ≻ *social* ≻ *environmental*, which indicates that it is imperative to enhance reporting practices in environmental dimension.

The governance dimension (7 %) received the highest score, followed by the educational dimension (4 %), the social dimension (2 %) and finally the environmental dimension (1 %). We calculate the scoring results of all sample HEIs and listed the 25 highest ranking HEIs in Table 3. 3 HEIs (Nanjing Medical University, National University of Defense Technology and Naval Medical University), giving no access to any information disclosure portal, receive no score, whereas other HEIs receive at least some points. Fudan University report most comprehensively with a total score of 84 points (19 %). Tsinghua University and Tongji University tie in second place with 66 points (15 %). All 3 HEIs focus on the educational dimension. The highest ranking HEIs show some regional trends: Of the 25 highest ranking HEIs, 20 HEIs or 80 % are located in Shanghai (10) or Beijing, and HEIs in Shanghai outperform those in Beijing by scoring high in the governance dimension; only 5 HEIs are from other regions and they were all of a different provincial-level administrative region (Chongqing, Jiangsu, Guangdong, Hebei and Anhui); Southwest University in Chongqing is the highest ranking HEI outside Beijing or Shanghai, with 18 points and ranked at the 15th position.

**Table 3:** Results of the four dimensions for the 25 highest ranking HEIs.

Ranking	HEI Name	EN	SO	GO	ED	Sum	Reporting level (%)
1	Fudan University	5	4	15	60	84	19
2	Tsinghua University	1	2	7	56	66	15
	Tongji University	1	2	16	47	66	15
4	Shanghai University	12	2	13	6	33	7
5	Peking University	9	5	11	7	32	7
6	Shanghai International Studies University	9	1	11	5	26	6
7	Shanghai Jiao Tong University	16	1	8	0	25	6
8	East China University of Science and Technology	11	2	11	0	24	5
	Shanghai Ocean University	3	2	14	5	24	5
10	Shanghai Conservatory of Music	7	2	13	1	23	5
11	Shanghai University of Finance and Economics	2	2	12	5	21	5
12	Donghua University	2	2	11	5	20	4
13	Renmin University of China	0	4	7	8	19	4
	East China Normal University	2	1	12	4	19	4
15	Southwest University	0	4	8	6	18	4
16	Beijing Jiaotong University	0	5	8	4	17	4
	Jiangnan University	0	2	9	6	17	4
	South China Normal University	0	2	10	5	17	4
19	Beijing Normal University	0	3	6	7	16	4
	Capital Normal University	0	2	7	7	16	4
	China Foreign Affairs University	0	3	8	5	16	4
	North China Electric Power University	0	3	8	5	16	4
	Hefei University of Technology	0	2	9	5	16	4
	China University of Mining and Technology, Beijing	0	3	8	5	16	4
	China University of Geosciences, Beijing	0	3	8	5	16	4
Maximum score		124	120	92	112	448	

### 4.7 Determinants of reporting

The regression results of (1) are presented in Table 4, which shows the determinants of reporting scores in each of the four dimensions and the overall score. Table 4 indicates the number of faculty members is significantly and positively associated with all reporting scores except the score of the environmental dimension. The number of students is positively related to all scores, but the relation is significant only with the environmental score and overall score. Funding indicates negative association with all scores, but the association is significant only for social and governance. *Expenditure* shows positive association with all scores, but association is significant only for environmental and social. For educational score, *Faculty* is the only significant regressor. Overall, the results suggest that the number of faculty members may be the most significant covariate associated with reporting scores in all dimensions except environmental.

**Table 4:** Determinants of reporting scores.

Variable	Reporting Score (Environmental)	Reporting Score (Social)	Reporting Score (Governance)	Reporting Score (Educational)	Reporting Score (Sum)
*Student*	0.030*	0.007	0.017	0.094	0.148*
	(0.017)	(0.008)	(0.016)	(0.071)	(0.075)
*Faculty*	−0.088	0.122**	0.213**	1.332***	1.579***
	(0.108)	(0.052)	(0.104)	(0.446)	(0.474)
*Funding*	−0.009	−0.007**	−0.010*	−0.004	−0.029
	(0.006)	(0.003)	(0.006)	(0.024)	(0.026)
*Expenditure*	0.010*	0.006**	0.008	0.002	0.026
	(0.006)	(0.003)	(0.006)	(0.024)	(0.026)
*Intercept*	−1.125	2.841***	5.792***	−4.566	2.942
	(1.508)	(0.72)	(1.446)	(6.228)	(6.615)
Province fixed effects	Yes	Yes	Yes	Yes	Yes
*N*	115	115	115	115	115
Adjusted *R*^2^	0.390	0.143	0.376	0.098	0.356

Note: *p < 0.1; **p < 0.05; ***p < 0.01. Standard errors are in parentheses.

## 5 Discussions

We note some new trends in ESG reporting by Chinese HEIs and discuss their managerial and policy implications as well. First of all, while ESG reporting has traditionally been regarded as a government-led activity [34] and the scope of information disclosure by MOE was defined by MOE regulation [40], we find 6 HEIs, all in Beijing and Shanghai, engaging in voluntary ESG reporting in standalone reports. The integration of an HEI's sustainability information by way of a single, reader-friendly report improves reporting quality and the readability of information and eventually acquiring legitimacy for the HEI. We note that the 6 HEIs with standalone sustainability reports ranked 1–5 and 7 in the final overall scores. Other HEIs in China are advised to consider adopting such best practices and integrating information that they already disclosed on their websites and social media accounts into a standalone sustainability report. We may draw some linkages between the current online contents and dimensions in the standalone sustainability report.

● Annual report (governance, environmental, educational)
● Information disclosure report (governance)
● Undergraduate teaching quality report (social, educational)
● Employment quality report (educational)
● Art education report (governance, educational)
● Faculty and union conference report (social)
● Academic committee report (social, educational)
● Graduate education quality annual report (social, educational)
● Scientific research results report (educational)
● Law-based governance report (governance)
● Energy and resource saving management report (environmental)
● Food safety report (governance)
● Charter (governance)
● Website content (governance, educational)
● Social media account content (governance, educational).

Second, the standalone sustainability reports almost invariably organized their information around the 17 SDGs of the United Nations, instead of adopting a more structured framework such as the GRI standards. SDGs are broadly defined goals with targets and indicators set at the country-level. It is therefore understandable why the standalone reports only loosely referred to the SDGs and did not specify any target or indicator. The disclosure according to SDGs therefore would depend largely on the HEIs’ own understandings of sustainability and does not facilitate uniform reporting practices nor evaluation and comparison of reporting quality. To encourage more streamlined and comparable ESG reporting practices, it is advised for Chinese HEIs to use a more indicator-based framework such as the GRI standards tailored to HEIs by adding an educational dimension. Reporting using a four-dimensional framework of indictors such as the one used in this study would facilitate Chinese HEIs to reach consensus over the meaning of ESG for HEIs and work towards a uniform ESG reporting framework for HEIs.

Third, we note that the sampled HEIs disclose the most information for the governance dimension, less for the educational and social dimensions, and the least for the environmental dimension. This is in contrast with the focus on the environmental goals in the sampled HEIs’ charters. It is imperative for Chinese HEIs to enhance their environmental reporting to live up to their environmental promises.

Fourth, the example of Peking University and Shanghai Jiao Tong University suggest that sub-HEI level entities such as business schools may have stronger incentives than HEIs to carry out ESG reporting and publish sustainability reports. Such sub-HEI level practices should be encouraged, especially because the management of many Chinese HEIs have yet to prioritize sustainability on their agenda. Such practices may help educate other members of the HEIs and serve as catalyst to trigger HEI-level actions of a later date.

Fifth, we see an opposite trend in some HEIs of disclosing less or no information or making online information no longer accessible to the public. For example, China University of Political Science and Law, after publishing its information disclosure report in 2020, no longer publishes such report on its portal in subsequent years. These practices might run afoul of the regulatory requirements and work against the needs of a broad range of stakeholders to monitor HEIs' ESG endeavors. Chinese HEIs may want to revisit the importance of information disclosure against the backdrop of sustainable development and consider the benefits of improved transparency for their stakeholders. MOE is also advised to engage in assessment and enforcement actions in terms of HEIs’ information disclosure performance and to amend the regulatory requirements by expressing adding sustainability-related information into the scope of disclosure.

It is apparent from the final ranking that HEIs in Shanghai and Beijing are generally forerunners among the sampled HEIs, leading in the educational and governance dimensions. This may be because local governments in these regions have additional ESG reporting requirements for HEIs. For example, Shanghai require education institutions, including HEIs, to enhance law-based governance with a defined set of indicators to evaluate the progress [59], and the law-based governance reports of sampled HEIs are released in response to such requirement. Local governments are therefore able to promote ESG reporting by adding relevant regulatory requirements.

## 6 Conclusions

We have analyzed the ESG reporting practices of 147 elite HEIs in China for the period 2020–2023. Our findings reveal that ESG disclosure is dispersed over different channels, including standalone sustainability reports, annual reports, charters, webpages, and social media platforms. Of the sampled Chinese HEIs, 4 % (6 of 147) publish 8 standalone sustainability reports, all of which are in Beijing or Shanghai. These standalone reports were released only recently (since 2021) and vary in terms of coverage (one to four dimensions), publisher, standard, length, collaboration, and external assurance. The 147 HEIs use a multiplicity of other online channels: about 600 annual reports of different types, 139 charters, websites of almost all HEIs (of which 105 with extensive relevant content), and 62 social media accounts. In general, the reporting is sporadic, fragmented and of a low quality. The results suggest that ESG reporting of Chinese HEIs is still at its early stage.

We also find that Chinese HEIs extensively organize the disclosure around the SDGs, without specifying any target or indicator. This is far from an ideal solution given that the SDGs are broadly defined for countries instead of the education sector. A more concrete and HEI-oriented reporting framework, like the one developed in this paper, is needed to improve the completeness, materiality, and comparability of information. Furthermore, we find the reporting quality across different dimensions is ordered as *governance* ≻ *educational* ≻ *social* ≻ *environmental*, underscoring the imperativeness to improve environmental reporting for Chinese HEIs. Also, the disclosure has a strong geographic pattern. Additionally, the number of faculty members is a more significant factor associated with reporting quality than number of students, funding, and expenditure.

There is a great potential for Chinese HEIs to improve ESG reporting, through systematic disclosure, usage of education-oriented disclosure framework, and more balanced reporting across different dimensions. While Chinese HEIs have made significant progress in global university rankings, their sustainability performance drags them down. For example, the QS World Rankings introduce sustainability as a new criterion in 2024 and none of the Chinese HEIs can break into top 200 on sustainability [60]. We believe that improving ESG reporting could help elevate the overall status and competitiveness of Chinese HEIs in the world.

Our study has several limitations. First, the 147 sampled HEIs are only a small portion (about 5 %) of all HEIs in China, although the sample is a normal size for content analysis of Chinese HEIs. We note that prior research on ESG reporting of Chinese HEIs use a sample of 44 or 70 HEIs [34,35]. The sampled HEIs represent the top HEIs in China in terms of academic performance, but their reporting quality does not necessarily reflect best practices in terms of ESG reporting. Neither does it necessarily reflect the sampled HEIs’ actual sustainability performance. Second, the selection of indicators might improve transparency and comparability but may harm the connection between the dimensions.

There are two avenues for future research. Based on the HEIs' disclosed information and information from other sources, it is possible to develop an ESG rating framework to evaluate the HEIs’ sustainability performance. Furthermore, ESG reporting at Chinese HEIs is still in its infancy and may change dramatically in coming years. It is valuable to continuously monitor the status of reporting in the future.

## Funding

This work is supported by the Fundamental Research Funds for the Central Universities (grant number 2023ZX002), the 10.13039/501100001809National Natural Science Foundation of China (grant number 71974201) and the Major Project of the National Social Science Foundation of China (grant number 22&ZD145).

## Ethical approval

As the present study was a secondary reanalysis of publicly available data, no ethical approval was required.

## Data availability statement

Data will be made available on request.

## CRediT authorship contribution statement

**Fei Mo:** Writing – review & editing, Writing – original draft, Formal analysis, Conceptualization. **Derek D. Wang:** Writing – review & editing, Visualization, Formal analysis, Conceptualization.

## Declaration of competing interest

The authors declare that they have no known competing financial interests or personal relationships that could have appeared to influence the work reported in this paper.
